# Midwestern Latino caregivers’ knowledge, attitudes and sense making of the oral health etiology, prevention and barriers that inhibit their children’s oral health: a CBPR approach

**DOI:** 10.1186/s12903-017-0354-9

**Published:** 2017-03-02

**Authors:** Kimberly K. Walker, E. Angeles Martínez-Mier, Armando E. Soto-Rojas, Richard D. Jackson, Sarah M. Stelzner, Lorena C. Galvez, Gabriela J. Smith, Miriam Acevedo, Laura Dandelet, Dulce Vega

**Affiliations:** 1 The Zimmerman School of Advertising and Mass Communications, University of South Florida, 4202 E. Fowler Ave, CIS1040, Tampa, FL 33620 USA; 2 Department of Cariology, Operative Dentistry and Dental Public Health, Indiana University School of Dentistry, 415 Lansing Street, Indianapolis, IN 46202 USA; 3 Department of Pediatrics, Indiana University School of Medicine, 5515 W 38th St., Indianapolis, IN 46254 USA; 4 Oral Health Research Institute, 415 Lansing Street, Indianapolis, IN 46202 USA; 5 Indiana University/Purdue University at Indianapolis, IN. 420 University Blvd, Indianapolis, IN 46202 USA; 6 La Plaza Inc., 8902 E. 38th Street, Indianapolis, IN 46226 USA; 7 Indiana University of Dentistry, Indianapolis, USA

**Keywords:** Community-based-participatory research, Latinos, Midwest, Child health, Focus groups, Oral health, Prevention

## Abstract

**Background:**

Using community-based participatory research, the Health Protection Model was used to understand the cultural experiences, attitudes, knowledge and behaviors surrounding caries etiology, its prevention and barriers to accessing oral health care for children of Latino parents residing in Central Indiana.

**Methods:**

A community reference group (CBPR) was established and bi-lingual community research associates were used to conduct focus groups comprised of Latino caregivers. Transcripts were analyzed for thematic content using inductive thematic analysis.

**Results:**

Results indicated significant gaps in parental knowledge regarding caries etiology and prevention, with cultural underlays. Most parents believed the etiology of caries was related to the child’s ingestion of certain foods containing high amounts of carbohydrates. Fewer parents believed either genetics/biological inheritance or bacteria was the primary causative factor. Fatalism negatively impacted preventive practices, and a clear separation existed concerning the perceived responsibilities of mothers and fathers to provide for the oral needs of their children. Females were more likely to report they were primarily responsible for brushing their children’s teeth, overseeing the child’s diet and seeking dental care for the child. Fathers believed they were primarily responsible for providing the means to pay for professional care. Perceived barriers to care were related to finances and communication difficulties, especially communicating with providers and completing insurance forms.

**Conclusion:**

The main study implication is the demonstration of how the CBPR model provided enhanced understanding of Latino caregivers’ experiences to inform improvements in oral prevention and treatment of their children. Current efforts continue to employ CBPR to implement programs to address the needs of this vulnerable population.

**Electronic supplementary material:**

The online version of this article (doi:10.1186/s12903-017-0354-9) contains supplementary material, which is available to authorized users.

## Background

With the growing Latino population, there is a need to better understand the personal, provider and system barriers that result in oral health disparities, especially in children [1]. Children immigrating from Mexico and those of Mexican descent born in the United States have higher dental caries rates and lower utilization of dental services than other American children of differing race and ethnicity [2].

Although multifactorial and complex, dental caries is preventable, yet prevention is largely dependent upon the composition of behaviors, attitudes and access to services [3]. In addition, the cultural beliefs of a race/ethnicity underlie these attitudes and behaviors, which ultimately influence the condition of the teeth and mouth through differences in diet, care-seeking behaviors and the identification of caries and its sequela. Cultural factors, therefore, have important implications for an individual's own health and their children’s health.

No less important to the prevention and treatment of dental carries is limited financial resources, lack of insurance and transportation, and a paucity of facilities having hours of operation. All have been identified as major barriers to oral care in the Latino population. Few of these focus on the growing population in the Midwest [4], even though the latest Census demographics indicate the Midwest experienced one of the largest growths [5]. Research on the oral healthcare needs of Latinos in the Midwest is limited, and further investigation of the way in which system barriers interact with an individual’s attitudes, knowledge and cultural beliefs to affect oral health care has been recommended [4, 6, 7].

This study employed focus groups comprised of Midwestern Latino parents to look at individual, cultural and system variables and their possible interactions using the health protection model (HPM). HPM is used to describe the multi-dimensional nature of individuals interacting with their environment to improve health and focuses on the interaction of individual experiences (including culture), cognitions, affects, and health behavioral outcomes for promoting health [8]. Specifically included in the analysis was an assessment of these interactions on the way Latino parents identified the etiology and prevention of dental caries and on their perceived barriers to accessing care for their children.

A community-based participatory research (CBPR) approach was used to gather the data in order to develop a culturally sensitive understanding of the population, given that the sample of Latinos interviewed are of the fastest growing segment of the population, who are primarily immigrants living below the poverty level without a barrio (an identifiable Latino neighborhood with more than 50% Latino inhabitants) for support [9]. A CBPR approach partners academics with community representatives in an equitable relationship to address health disparities. The relationship allows the participants to create social networks to better understand linkages between culture, research design, analysis and interpretation [10]. Previously, most studies assessing the causes for health disparities in Latino Americans have not engaged community partners. As a result, they may not have had access to information concerning important variables, including local economic, social and behavioral factors that could potentially affect oral health.

The present study explored a sample of Midwestern Latino caregivers’ understanding and experiences of oral health care for their children. To our knowledge, this is the first study to investigate the multi-causal reasons for disparities in the oral health care of Midwestern Latino children, using a CBPR approach.

### Aims

The primary aim of the present investigation was to utilize CBPR to explore how Latino caregivers understand and make sense of good oral health for their children. Ultimately, the aim was to develop a culturally sensitive educational model and intervention for addressing the oral healthcare needs of Central Indiana Latino parents who utilize the services of La Plaza for their children.

The research questions that guided this study were:

1. What are the experiences (including culture), cognitions, and affects related to caries etiology, prevention and management and perceived barriers to accessing oral healthcare for Latino caregivers’ children?
2. How do the experiences, cognitions and affects (emotions) translate to possible behavioral outomes?
3. Based on results, what is an educational model for educating and improving the oral health behaviors and decision making of Latino caregivers to improve the oral health of their children?

## Methods

The research team consisted of faculty and staff from the Indiana University School of Dentistry (IUSD) and the La Plaza Latino Community Organization (LCO), both located in Indianapolis IN, USA. IUSD and the LCO have a 10-year history of collaboration on research and service projects. La Plaza’s missions are to advocate for and prepare Latino students for academic success and to connect Latino families with local health and social services through their network of community partners. LCO members are bilingual and have experience providing culturally and linguistically appropriate services.

Early on, the IUSD and LCO realized they had a shared interest in forming a CBPR group to address the high prevalence of oral disease and the largely unmet need for oral services in this community. The partnership was important to the success of the investigation in three ways. First, the LCO members were familiar with the oral needs of the Latino community and, as a result, were able to identify research questions relevant to the community. Secondly, they provided a community advisory board (CAB) that assisted in developing and conducting the project; and finally, they formulated a recruitment plan to identify possible participants. As co-investigators, the LCO members were active in all phases of the pilot and main investigation, including panelist recruitment, consenting data collection, and debriefings.

In order to identify the factors that influence the oral health of Latino parents’ children, a qualitative research infrastructure consisting of focus groups with Latino parents was agreed upon. The research team of academics and the LCO members developed a semi-structured focus questionnaire to identify levels of understanding and beliefs concerning the etiology and prevention of caries, with an emphasis on determining how the parents perceived the importance of oral hygiene, sealants, fluoride and food choices, all of which are affected by social and cultural understanding [11]. Questions were also formulated to ascertain the barriers the parents believe impeded access to oral health services. All questions were piloted using a small focus group to ensure they were understandable and would elicit open discussion (Additional file 1).

Prior to recruitment of the parents for the focus groups, the protocol and supporting documents were approved by the Institutional Review Board (IRB) of Indiana University (IRB # 062-51). Parents were recruited using a two-stage process. First, the LCO identified recent attendees of La Plaza who they knew needed dental care. Second, advertisements describing the study were aired in Spanish on the community radio station (*Radio Latina*) asking interested persons to participate. Inclusion criteria were that individuals self-identified as being of Latino origin, were willing to provide written informed consent, were willing to participate in a focus group, and be a parent of a child who had received a dental examination in a parallel research study [12]. Potential panelists were excluded if they could not meet the inclusion criteria or were unable to be scheduled to participate in one of the focus groups.

Two moderators conducted eight focus groups over a 4-month period. All of the one-hour sessions were conducted in Spanish at La Plaza with 14–18 participants in each. Although the groups were larger than expected due to perceived issue importance, trained moderators assured all voices were heard, using techniques such as direct questioning, asking for differences of opinion, and encouraging participants to piggyback off of ideas [13]. A written verbatim transcript was made from an audio tape recording and written notes taken by the investigators. The transcripts were translated into English and reviewed to ensure they were complete and accurate. Participants completed a demographic questionnaire concerning their age, gender, duration of residence in the US, number and age(s) of their children, and employment status. Each participant received a small compensation.

Two researchers collaboratively coded the data together using the constant comparative method to uncover themes related to the parents’ perceptions of the aforementioned knowledge, beliefs, and barriers. Constant comparison analysis is especially relevant when multiple focus groups are used within the same study. In constant comparison analysis, data are analyzed by individual group, allowing the researcher to assess if themes that emerged from one group also emerged from other groups. Thus, researchers can test the themes and determine when theoretical saturation occurs (e.g. the point in which no new conceptual insights are generated, evidencing justification for conceptual categories) [14]. Constant comparison is considered a form of triangulation that improves reliability because the views and experiences of respondents are continuously compared for consistency [13]. Reliability was also ensured in this study with the use of multiple coders, review and agreement of coding results by La Plaza team members, and feedback loops, whereby the moderator feeds back what has been learned into the questions asked [13].

## Results

### Demographic characteristics

There were 130 participants; (100 Female, 30 Male) ranging in age from 18 to 54 with the majority being 24–34 years-of-age. All were low SES, based upon their screening for La Plaza services, which requires families to have income below the federal poverty level (See Federal Poverty Guidelines). (See Table 1 for demographics and example of corresponding quotes).

**Table 1:** Demographics and associated quote examples

Gender						
Male	30					
Female	100					
Gender total	130					
Age						
18–23	31					
24–34	68					
35–44	19					
45–54	12					
Age total	130					
Employed						
Yes	60					
No	70					
Total employment	130					
Reside in U.S.						
1–2 years	3					
3–6 years	10					
+ 6 years	117					
Total reside in U.S.	130					
Number of children per household	Range = 1–5 Mean = 3					
Age of children	Range = 1.8 mths–13 Mean = 8.2 years					
Comment	Gender	Age of subject	Number of subject's children	Is subject employed?	Subject's employment	Time the subject has been living in the U.S.
“Infections happen when teeth are new because they (children) walk with friends or other children (and) because they share toys or candy.”	Female	18–24	1–2	Yes	Restaurant	<1 Year
“There are mothers who breastfeed but do not like it because they say your breasts drop. So they give them milk from the store and think that makes them benefit their children because milk now comes with vitamins; but the power of the mother is also best for the child.”	Female	18–24	1–2	Yes	Supermarket store	3–5 Years
“The child is so small, so his teeth cannot be washed properly.”	Female	25–34	3–4	Yes	Cleaning service	2–3 Years
“(I brush his teeth) maybe once or three times a week (when having) a mass of food buildup”.	Female	25–35	1–2	Yes	Restaurant	2–3 Years
"I took his card and used his insurance. I did this because without insurance I could not afford it and I had to think about the responsibility I have towards my wife and kids."	Male	18–24	3–4	Yes	No answer	1–2 Years
"We do not speak the language. If we have a problem and they (teeth) start to hurt, we cannot go to the dentist because of how we feel inhibited because we cannot speak English, not properly."	Male	18–24	3–4	Yes	Construction	1–2 Years
"I stay with the same dentist and he told me that I could change all my fillings so that the teeth do not look like they had fillings. But he said I would not recommend because the amalgams had more resistance. So even if they look ugly at least I'm healthy. Much depends on the doctor."	Male	18–24	3–4	Yes	Discount store	1–2 Years

### Themes

#### Etiology

- Linear as a result of diet
- Identification post-caries development

#### Prevention

- Mother’s right to choose breastfeeding
- Mother’s responsibility for diet and hygiene
- Fatalism
- Lack of knowledge of hygiene and protective behaviors

#### Barriers

- Financial/transportation
- Language

### Etiology and prevention

In reviewing the transcripts, diet was a central variable affecting parents’ sense making of the etiology of caries and prevention behavior(s). Overall, caries etiology was not well understood by parents, as none described the etiology as a complex causal system. Instead, most described it as a linear relationship between the frequent ingestion of high carbohydrate foods and beverages (including soft drinks) and the development of carious lesions. Less commonly, genetics or bacteria were identified as possible factors, although some mothers believed that if the etiology of caries was microbial, then it was transmissible. As one mother stated:

“Infections happen when teeth are new because they (children) walk with friends or other children (and) because they share toys or candy.”

In addition, the primary indicators for the presence of caries were believed to be the development of staining (yellow or other discoloration) and dental pain. Some stated oral malodor and/or gingival bleeding would be present. Contrarily, some believed gingival bleeding indicated health and adequate brushing.

In terms of prevention, there was considerable confusion among mothers as to the role of milk in contributing to or preventing caries. Inadequate intake was believed to result in poor tooth development and the likelihood for caries development, yet many mothers questioned the amount considered “inadequate”. They were also unsure if the form (whole versus skim) ingested might be beneficial or detrimental.

Opinions concerning a mothers’ right to choose between breastfeeding and cow’s milk was a strongly held belief that outweighed discussion of the health benefits of breastfeeding, although a few mothers voiced the opinion that cow’s milk may be more advantageous because it is vitamin-fortified.

“There are mothers who breastfeed but do not like it because they say your breasts drop. So they give them milk from the store and think that makes them benefit their children because milk now comes with vitamins; but the power of the mother is also best for the child.”

Fatalism was a common belief, as very few parents believed caries could be prevented in their children, so little value was placed upon caries prevention. This was in contrast to the belief held by many parents that “children who are born in the U.S. have privileged gums.”

Because fatalism is correlated with lower oral health knowledge and behaviors, strong fatalistic beliefs toward their child’s inevitable caries development could have affected the knowledge of their children's oral health needs, supervision of their child’s hygiene regimen and seeking of dental care. Both mothers and fathers in this sample lacked knowledge of accepted oral hygiene practices and the preventive needs of their children. Most participants were not familiar with the role of fluoride beyond a general sense that it was protective for teeth, and almost none were aware that fluoride is added to tap water. They were unsure of the recommended brushing frequency or duration, with responses ranging from one to four times daily and from 30 s to 4 min. They also were unsure of the appropriate time to identify a dental home for their child. None were aware of the role of sealants, although 56% of their children have sealants [12]. Many parents did not know to brush the teeth after a bottle is given at bedtime. Many mothers acknowledged they did not routinely perform oral hygiene on their children’s teeth, assist them with toothbrushing or supervise their brushing. As two mothers stated,

“ The child is so small, so his teeth cannot be washed properly.”

“ (I brush his teeth) maybe once or three times a week (when having) a mass of food buildup”.

**Fig. 1:**
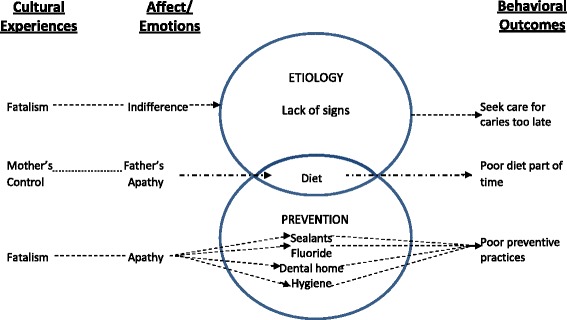
HPM variables for etiology and prevention. In Fig. 1, the middle descriptions represent the main cognitive themes encompassing etiology (lack of knowledge of signs) and prevention (lack of knowledge of sealants, fluoride, dental home, hygiene). It demonstrates that knowledge about nutrition overlaps both etiology and prevention. The lines to each cognitive theme show our particular data findings related to the HPM constructs of culture and affect and how they affect behavior outcomes (represented as lines away from *circles*). Here, fatalism (culture) and indifference (affect) lead to decreased cognitive recognition of oral disease, leading to less dental access and care. Mothers’ control over children’s health (culture) and father’s apathetic state (affect) affect understanding of controlling children’s diet (cognition), leading to poorer children’s nutrition when fathers care for children. Fatalism (culture) and apathy (affect) affect understanding of preventive practices, leading to poor oral preventive practices

For those few parents who believed caries preventable, a good diet and taking vitamins were thought to be important, and the responsibility for these tasks was believed to be primarily the mother’s. Mothers commonly felt that it was their responsibility, rather than the fathers', to "control" their child's diet, and that if the "father is watching, the child will eat anything." Some mothers emphasized that they monitor sugar intake at home because they believed their children ingest too much sugar at school. However, most were not aware of the role of sweeteners and fermentable carbohydrates on erosion. A conceptual graph describing how the HPM constructs of cognitions, affect and cultural experiences affect parents’ sense making of the etiology of oral disease and preventive practices for their children is seen in Fig. 1. The findings represent the main themes expressed about each construct from focus group discussion.

### Perceived barriers

#### Finances, amenable facilities and transportation

The most commonly cited barrier to providing professional oral care for their children was affordability. Many parents stated that dental care is expensive and they lacked the necessary income or insurance to provide regular dental care for their children, despite being aware of its importance. Fathers clearly voiced a cultural responsibility to provide financially for their child's healthcare. Many stated they had neglected their own oral health to provide care for their child. One father reported using another person’s identification in order to provide treatment for his children.

“I took his card and used his insurance. I did this because without insurance I could not afford it and I had to think about the responsibility I have towards my wife and kids.”

Lack of availability of services and hours amenable to many of the parents’ schedules, given nontraditional working hours and transportation, were also cited as perceived barriers. As a result of the lack of locally available services, individuals believed a low cost community mobile clinic, especially if it could set up in a local school or community center, would increase their ability to obtain treatment.

### Communication/health literacy

Many felt their limited English proficiency made it difficult communicating on the telephone or discussing their concerns with a dentist, and it impeded their ability to understand and complete insurance forms. As a result, parents often did not seek regular care for their children.

“We do not speak the language. If we have a problem and they (teeth) start to hurt, we cannot go to the dentist because of how we feel inhibited because we cannot speak English, not properly.”

Language and cultural differences also negatively impacted some parents' active participation in their own health, as they often kept the locus of power in the dentists' hands. As one father stated,

“I stay with the same dentist and he told me that I could change all my fillings so that the teeth do not look like they had fillings. But he said I would not recommend because the amalgams had more resistance. So even if they look ugly at least I'm healthy. Much depends on the doctor.”

**Fig. 2:**
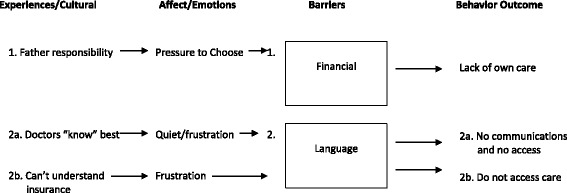
HPM variables for main barriers. In Fig. 2, the middle squares represent the main cognitive barriers of finances and language. The lines to each barrier show how our particular data findings related to culture and emotions affect behavior outcomes (represented as lines away from *squares*). Here, fathers’ perceived financial responsibility (culture) and feelings of having to choose who gets oral care (affect) affect financial barriers that can lead to the behavioral outcome of poor oral care for either themselves or their children. Belief that dentists are in control and lack of comprehension of insurance (culture/experience) combined with frustrations (affect) affect communication barriers and access (outcomes)

Figure 2 shows a conceptual graph describing how the main themes under the HPM constructs of cognitions, affect and cultural experiences, described by focus participants, affect the oral health behaviors of their children for each barrier.

## Discussion

This study demonstrates that the sense making of the etiology of oral disease, access to oral care, and preventive oral behavior practices occur within a complex relationship among environment barriers, cultural beliefs, cognitions and attitudes. Results of the study represent an initial project in what should be a long collaborative experience, as much work remains to improve the oral health of Latino youth utilizing the services of La Plaza. Continuing to use a CBPR approach, and now having the confidence of the community and supporting data, the team of dental researchers and the CLO have set four objectives based on study results:

- The need for education materials that are culturally and linguistically appropriate
- The need for insurance information that is conveyed in understandable terms
- The need for providers who are culturally sensitive to Latino needs.
- The need for services and transportation to improve access to care

**Fig. 3:**
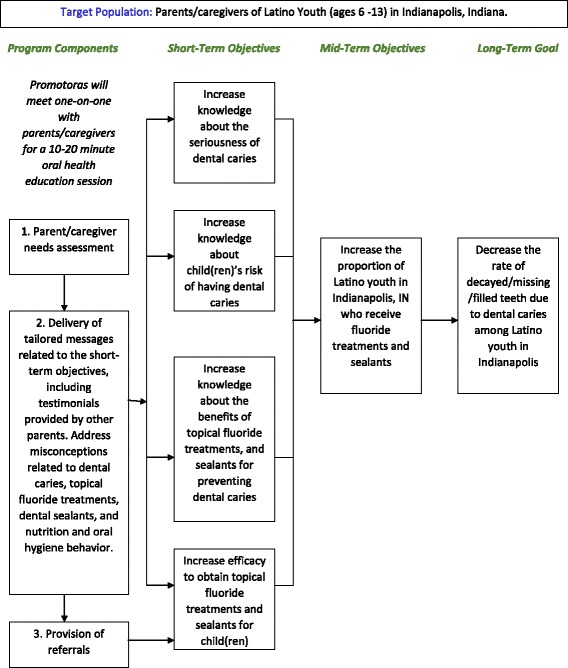
Program education intervention model

As the primary objective of La Plaza is health education, the team prioritized education and the development of an educational program intervention, as the first collaborative step toward the long-term goal of reducing the rate of dental caries in Latino youth in Central Indiana. Figure 3 shows the Program Education Intervention Model. The program model proposes education sessions with Latino parents who utilize La Plaza services, with the long-term objective of improving the oral health of young school age children (6–13 who can receive services), although education is also targeted at care for younger children (e.g. bottle feeding, start of dental home). The sessions are to be led by “promotoras”, who are community Hispanic leaders, and are culturally tailored to be sensitive to the needs of the population, as identified in our study. A toolkit to deliver the messages has already been developed, encompassing recruitment letters for the promotoras, a User’s Guide to Oral Health for them as well as participants, oral health brochures and pamphlets, sealant pictures and explanations, and instructions on oral hygiene care and standards.

The tailored messages in the toolkit are designed to target the many misconceptions related to dental caries, as our study results clearly witnessed most of the parents believed a single factor (diet) was responsible for caries development, as is consistent with previous findings [2, 15]. Because caries is multifactorial, greater attention must be made to educate parents as to how these factors interact to cause or prevent the disease over time and emphasize the disease is preventable and not inevitable. Research has shown that fatalistic beliefs of parents are correlated with having less knowledge of their children's oral health needs, less supervision of their child’s hygiene regimen, less seeking of dental care, and more frequent ingestion of high carbohydrate beverages at bedtime [16], all which were mirrored in this study. Overcoming the feeling of fatalism, therefore, is important if we are to lower the prevalence of disease in this population. Increasing self-efficacy to obtain topical fluoride treatments and sealants for preventing dental caries is one way we address overcoming fatalistic views.

Nutrition is also emphasized in our education materials and program. Many mothers interviewed were unsure of the properties (whole or skim) most conducive to oral health, the quantity to provide, and whether an infant is better served by store or breast milk. Education is directed toward explaining the difference between whole and skim milk and the role of calcium in tooth development. Although some mothers felt they were better at monitoring sugar intake at home than at school, there was still a lack of clarity of what is included as a sugary substance. For this reason, education also emphasizes that frequent ingestion of sugar-containing beverages causes caries, especially if the child is allowed to fall asleep without cleaning the teeth. Parents are also educated that fermentable carbohydrates are found in many foods that they may not recognize as being high in carbohydrates.

Education also addresses establishment of a dental home by age one in conjunction with information about the role of fluoride, sealants and early initiation of a regular oral hygiene regimen under supervision as important preventive measures, as Latino mothers in this study lacked knowledge concerning when to initiate brushing, when to allow the child to brush without supervision, the recommended brushing techniques and frequency and duration of brushing. Education is aimed at both genders to attempt to even the responsibility for diet and oral hygiene practices, as research shows that more frequent brushing occurs with children under two-parent guidance [17].

In addition to the education program, an additional goal is to develop training modules to address the cultural and language barriers uncovered in this study that frustrate the parents’ oral health experience. Of importance are the development of training sessions related to understanding insurance documents, common oral terminology, accessing online dental information and confidence-building sessions for communicating with providers. Sessions in which Latino parents can practice negotiating effective communication, ranging from the initial telephone appointment request to the comprehension of insurance forms to communicating with the dentist were identified as substantial barriers to accessing oral care for their children. Ultimately, the improvement in language and communication skills can help empower parents to develop an internal locus of control toward protecting their children’s oral health [18, 19]. Development of more oral health services with providers who are sensitive to the needs of the population are long-term goals. Future collaborative research of local providers of oral health in the community is desirable to determine willingness to collaborate and train.

### Limitations

There are several limitations that need to be acknowledged. First, although focus groups allow in-depth information to be obtained, caution must be exercised when extrapolating results to other Latino populations. However, the HPM framework that guided this study provides concepts that are generalizable; namely that individuals interact with their environment to improve their health. These interactions include an individual’s cultural experiences, cognitions, and affect. Healthcare professionals are an essential element of the environment, and those professionals who can influence positive experiences and affect can improve the likelihood that protective dental behaviors are exhibited [8]. Secondly, more women than men participated, and some responses could reflect the distribution of gender and cultural norms present. Data is also qualitative in methodology, so different perspectives may still be desirable in quantitative contexts. Last, our panel did not contain many recent immigrants, and thus responses from more recent immigrants may differ. It would be beneficial to explore opinions of those more recently integrated, as those who are may feel even more estranged from the community and potentially view more hardships caring for their children’s oral health.

## Conclusions

The participatory approach of this study enabled a comprehensive description of the issues involved in Central Indiana Latino parents’ sense making of oral health and barriers to providing adequate oral health care for their children. The involvement of parents, researchers and community advocates in this project provided a snapshot of the importance of cultural understanding along with financial barriers in addressing improvements.

There are many opportunities to continue working together for the future of this vulnerable Latino parent/child population, as nationally, there is a shortage of Hispanic and Latino dental professionals to provide the culturally sensitive experiences needed for this growing population [20]. Change is being affected by using promotoras to help deliver the next stage of education, which dovetails with the national concerted effort to help reduce social, political and economic pressures of the dental profession to meet diverse health needs. Our education model/intervention for providing culturally sensitive assistance to Latino parents at La Plaza can be expanded to reach beyond our original target group to help reduce the barriers to oral health care that affect the growing population of Latino immigrant children in the Midwest.

## Additional file

Additional file 1: Focus group questions. Additional file 1 shows leading focus group questions in the three categories of caries etiology, prevention and barriers to oral care. (DOCX 76 kb)

## Abbreviations

CAB: Community advisory board
CBPR: Community-based participatory research
HPM: Health protection model
IRB: Institutional Review Board
IUSD: Indiana University School of Dentistry
LCO: Latino community organization
