# Supplementary material for: Midwestern Latino caregivers’ knowledge, attitudes and sense making of the oral health etiology, prevention and barriers that inhibit their children’s oral health: a CBPR approach
Source: BMC Oral Health. 2017 Mar 2;17:61. doi: 10.1186/s12903-017-0354-9 (PMC5335721; doi:10.1186/s12903-017-0354-9)
Supplement: Additional file 1: — Focus group questions. Additional file 1 shows leading focus group questions in the three categories of caries etiology, prevention and barriers to oral care. (DOCX 76 kb) [file 12903_2017_354_MOESM1_ESM.docx]

# Additional File

# ­­­­­­­­­­Sample focus group questions of experiences with caries etiology, prevention and barriers to oral health.

# Caries Etiology

# How do you know when your child has a cavity?

# What causes cavities?

# How knowledgeable do you feel about your child’s oral health? About cavities?

# How do you feel about the ultimate oral health of your children (and in terms of caries, especially)?

# When do you typically seek care for your child’s cavities?

# Prevention

# How can tooth decay in your children be prevented?

# How often should your children brush their teeth?

# (Follow up---How long should they brush each time?; How often do they brush?

# What are the hardships, if any, to getting them to brush regularly?;

# How often do you oversee their brushing, if you do?

# Have your children received sealants? (follow up with Do you know what sealants are?)

# What is the role of fluoride?

# When should a child establish a dental home? When did or will your child establish a dental home?

# Barriers

# What, if any, are the difficulties you face when you seek dental care for your children?

# Follow up: Based on the difficulties:
